# Effects of decreasing the out-of-pocket expenses for outpatient care on health-seeking behaviors, health outcomes and medical expenses of people with diabetes: evidence from China

**DOI:** 10.1186/s12939-022-01775-5

**Published:** 2022-11-16

**Authors:** Wenwen Du, Ping Liu, Wei Xu

**Affiliations:** School of International Pharmaceutical Business, China Pharmaceutical University, Nanjing,, 211198 Jiangsu China

**Keywords:** Decreasing the out-of-pocket expenses for outpatient care, Health-seeking behaviors, Health outcomes, Medical expenses, Diabetes patients

## Abstract

**Background:**

To improve access to outpatient services and provide financial support in outpatient expenses for the insured, China has been establishing its scheme of decreasing the out-of-pocket expenses for outpatient care in recent years. There are 156 million diabetes patients in China which almost accounts for a quarter of diabetes population worldwide. Outpatient services plays an important role in diabetes treatment. The study aims to clarify the effects of decreasing the out-of-pocket expenses for outpatient care on health-seeking behaviors, health outcomes and medical expenses of people with diabetes.

**Methods:**

This study constructed a two-way fixed effect model, utilized 5,996 diabetes patients’ medical visits records from 2019 to 2021, to ascertain the influence of decreasing the out-of-pocket expenses for outpatient care on diabetes patients. The dependent variables were diabetes patients’ health-seeking behaviors, health outcomes, medical expenses and expenditure of the basic medical insurance funds for them; the core explanatory variable was the out-of-pocket expenses for outpatient care expressed by the annual outpatient reimbursement ratio.

**Results:**

With each increase of 1% in the annual outpatient reimbursement ratio: (1) for health-seeking behaviors, a diabetes patient’s annual number of outpatient visits and annual number of medical visits increased by 0.021 and 0.014, while the annual number of hospitalizations decreased by 0.006; (2) for health outcomes, a diabetes patient’s annual length of hospital stays and average length of a hospital stay decreased by 1.2% and 1.1% respectively, and the number of diabetes complications and Diabetes Complications Severity Index (DCSI) score both decreased by 0.001; (3) for medical expenses, a diabetes patient’s annual outpatient expenses, annual inpatient expenses, annual medical expenses and annual out-of-pocket expenses decreased by 2.2%, 4.6%, 2.6% and 4.0%; (4) for expenditure of the basic medical insurance funds for a diabetes patient, the annual expenditure on outpatient services increased by 1.1%, and on inpatient services decreased by 4.4%, but on healthcare services didn’t change.

**Conclusion:**

Decreasing the out-of-pocket expenses for outpatient care appropriately among people with diabetes could make patients have a more rational health-seeking behaviors, a better health status and a more reasonable medical expenses while the expenditure of the basic medical insurance funds is stable totally.

**Supplementary Information:**

The online version contains supplementary material available at 10.1186/s12939-022-01775-5.

## Background

According to statistics from the World Health Organization (WHO), about 15 million people die of diabetes every year around the world [1]. China has the largest number of people with diabetes globally, up to 156 million people, approximately a quarter of the world’s total [2, 3]. Between 1990 and 2017, China’s all ages years of life lost (YLL) from diabetes increased by 55% and the ranking of diabetes rose from 19th to 8th in the causes of disability-adjusted life-years (DALYs) [4]. Diabetes cannot be cured, making its treatment relies on taking medicines and having regular examinations, which could result in a heavy economic burden for individuals in the long term. In 2021, the per capita health expenditure of people with diabetes in China were $1,173.5, approximately 21% of the per capita income in the same period [5]. Outpatient services plays a crucial role in diabetes daily management and is also good at reducing the incidence or severity of diabetes complications [6–8]. Therefore, improving access to outpatient services and providing financial support in outpatient expenses for diabetes patients has become an important goal in China and elsewhere.

Limited by the inadequate pooled funds at the early times, the Chinese social health insurance system (SHI) gave priority to reimbursing the insured people with major or serious illness [9, 10] which could bring a heavy economic burden and disease burden to patients and society like cancer, cirrhosis, heart failure and renal failure [11–13], especially in their hospitalization expenses while ignoring the financial support for outpatient services [14, 15]. However, comparing with the inpatient services, the outpatient care has a higher utilization frequency and could meet the most of the healthcare needs in many instances [16], such as primary health care services, daily follow-up of chronic diseases and health education to patients [17, 18]. The low reimbursement for outpatient expenses brings about a series of problems such as restraint in the demands for healthcare and occurrence of irrational hospitalizations in China [19, 20]. Faced with these emerging situations, in 2007, the Chinese federal government proposed that regions where conditions allow should set up a scheme of decreasing the out-of-pocket expenses for outpatient care. To alleviate the financial burden on urban and rural residents with diabetes and hypertension who were insured under the basic medical insurance system, the Chinese government decided to pay for their expenses of drugs that belong to the National Reimbursement Drug List (NRDL) provided through outpatient services in October 2019. But detailed reimbursement policies were made by regions according to their actual situation. In April 2021, China issued a guideline on improving the mutual-aid mechanism for covering outpatient bills under its basic medical insurance system for the employed, with the aim of exploring a pooling mechanism for costs of general outpatient services for chronic and common diseases. Overall, China is now accelerating the establishment of the scheme of decreasing the out-of-pocket expenses for outpatient care, which could provide more access to outpatient services for the insured.

Theoretically, an appropriate out-of-pocket expenses for outpatient care could make people with diabetes have rational health-seeking behaviors and good health outcome, as well as realize the efficient allocation of medical resources. However, whether this is the case has always been controversial. On the one hand, a number of studies found that decreasing the out-of-pocket expenses for outpatient care could encourage patients to utilize more outpatient services and satisfy their needs for healthcare. In this progress, the previous underuse of outpatient services will be filled up, which in turn transfers some inpatient treatment to outpatient visits, particularly the unnecessary hospitalization. Finally, the rationality of patients’ health-seeking behaviors will be promoted [21, 22], especially among the insured in low-income and low-education regions and those with chronic diseases such as hypertension and diabetes [23]. As is known to all, taking full advantage of primary healthcare that mainly provides outpatient services could greatly improve individuals’ health outcomes for some diseases like diabetes [24]. After decreasing the out-of-pocket expenses for outpatient care, patients have more access to healthcare services, allowing them enjoy a better and more systematic disease management as well as a healthier status [25]. Owing to this, patients’ utilization of inpatient services and even overall healthcare services declines, which ultimately helps achieve the goal of reducing expenses and saving basic medical insurance funds [26]. On the other hand, some scholars believe that it’s not worthwhile to decrease the out-of-pocket expenses for outpatient care. Under many circumstances, especially when the disease is more severe, the treatment provided by outpatient services is indeed limited [27]. Meanwhile, for patients in regions that are not equipped with productive medical staff and equipment, over-reliance on outpatient services is likely to result in missing the best chance to intervene in the disease, which could cause further damage to health [28]. Some studies have shown that excessive outpatient coverage can easily induce people to overuse outpatient services, while making no sense to the improvement in health, even bringing about waste of medical resources [29]. Because of these inconsistent conclusions, it is still meaningful to systematically clarify the effects of decreasing the out-of-pocket expenses for outpatient care on the health-seeking behaviors, health outcomes and medical expenses among people with diabetes [21, 30, 31], which will perfect China’s basic medical insurance system and make expenditure good value for money.

Based on a unified administrative medical insurance claim dataset that includes all detailed information of enrollees’ healthcare services, this paper tries to answer three questions by constructing a two-way fixed effect model: (1) Will decreasing the out-of-pocket expenses for outpatient care affect diabetes patients’ health-seeking behaviors, and what is the relationship between them? (2) How will diabetes patients’ health outcomes change along with decreasing the out-of-pocket expenses for outpatient care? (3) What’s influence of decreasing the out-of-pocket expenses for outpatient care on diabetes patients’ medical expenses?

## Data and methods

### Changes in policy and data overview of the sample region

#### Changes in policy of the sample region

Before December 2019, the scheme of decreasing the out-of-pocket expenses for outpatient care under the basic medical insurance system for urban and rural residents formulated by Nanjing, Jiangsu Province, China was as follows [32]: all outpatient expenses falling within the coverage of the reimbursement that caused by the insured may get paid via the basic medical insurance funds according to a unified standard. The deductible of outpatient expenses is $31.70^1^. For people treated in community health service centers, the pooled funds would cover 50% of their outpatient expenses, otherwise 30%. The annual reimbursement cap line for outpatient services was $47.54. For residents over 80 years old, the coverage percentage of outpatient expenses paid by pooled funds increased by 5% while the annual reimbursement cap line for outpatient services increased by 10% on the basis of the above regulations.

After December 2019, Nanjing developed a separate scheme of decreasing the out-of-pocket expenses for diabetes outpatient care on the basis of the original system aforementioned [33]. In the new policy, for people with diabetes, their annual reimbursement cap line for outpatient services is $126.78; for people with diabetes and hypertension at the same time, their annual reimbursement cap line for outpatient services is $190.17. The deductible and coverage percentage of the outpatient expenses are consistent with original system.

In conclusion, the new policy actually improves the reimbursement cap line while remains others unchanged. Under such circumstances, the out-of-pocket expenses for outpatient care of people with diabetes decrease and their financial burden caused by outpatient services could get relieved eventually.

#### Details of sample data

The Chinese Healthcare Security Administration was found on May 31st, 2018, which has promoted the development of the construction of medical insurance claim dataset in China greatly. On account of the accuracy and integrality of statistics, this paper chooses to utilize detailed information concerning enrollees’ healthcare services from 2019 to 2021. Finally, a total of 5,996 diabetes patients in the sample region were fully included in this study. All their visits information from 2019 to 2021 was used as sample data for analysis, which could avoid retrospective bias due to the use of survey data or sampling data in previous studies and help improve the accuracy of the study results. The set of sample data included all the medical visits records of both outpatient services and inpatient services utilized by sample population within three years, whose amount was 501,854 records, including 494,251 outpatient records and 7,603 inpatient records. Each record contained individual information (gender, age, registered household type), medical institution information (name, level, and type of institution), medical visit information (outpatient service or inpatient service, admission and discharge date, disease diagnosis and disease code) and expenses information (total medical expenses, outpatient expenses, inpatient expenses, out-of-pocket expenses, expenditure of the basic medical insurance funds).

### Study model

#### Individual-time two-way fixed effect model

Since the sample data were panel data, this study used the Hausman test to determine whether the random effect model or the fixed effect model is the most appropriate choice for the empirical analysis [34]. The results showed that the p-value corresponding to the Hausman test is significant at the 1% level, which indicates that the fixed effects model should be chosen. Additionally, there were also “individual effects” that do not vary with time and “time effects” that do not vary with individual heterogeneity in this study. Therefore, the individual-time two-way fixed effect model was finally adopted as the empirical model for this study. The model was set as follows:

Y_i_ = β_0_ + β_1_X + β_2_U_i_ + µ_t_ + η_i_ + ε_it_ (1).

Where Y_i_ is the dependent variable, including diabetes patients’ health-seeking behaviors, health outcomes, medical expenses and expenditure of the basic medical insurance funds for them; X is the core explanatory variable, representing the out-of-pocket expenses for outpatient care; U_i_ is the set of control variables, including a series of related factors that affect the dependent variables, such as age and whether using the medical services for other diseases; µ_t_ and η_i_ are dummy variables for year and individual, respectively, used to control the time and individual effects; ε_it_ is the random noise term; i denotes different individuals, and t denotes time.

Since the values of above variables are positive and different widely, in order to provide an easy calculation and eliminate heteroscedasticity caused by different data dimension, the expense-related indicators (medical expenses, expenditure of the basic medical insurance funds) and the length of hospital stays were subject to natural logarithm processing.

#### Variable selection

##### Dependent variables

The dependent variables (Y_i_) in this study included diabetes patients’ health-seeking behaviors, health outcomes, medical expenses and expenditure of the basic medical insurance funds for them. See Table 1 for specific indicators of dependent variables and their descriptions.

**Table 1:** Description of dependent variables

Variable		Definition
Health-seeking behaviors	Annual number of outpatient visits (Y_1_)	Total number of outpatient visits of a diabetes patient per year
	Annual number of hospitalizations (Y_2_)	Total number of hospitalizations of a diabetes patient per year
	Annual number of medical visits (Y_3_)	Total number of medical visits of a diabetes patient per year, which is equal to annual number of outpatient visits plus annual number of hospitalizations
Health outcomes	Annual length of hospital stays (LnY_4_)	Total days spent on hospitalization by a diabetes patient for any cause per year
	Annual average length of a hospital stay (LnY_5)_	Average days spent on a single hospital stay by a diabetes patient for any cause per year
	Number of diabetes complications (Y_6_)	Number of diabetes complications suffered by a diabetes patient
	DCSI score (Y_7_)	Diabetes Complications Severity Index (DCSI) score is a total score for diabetes complications of a diabetes patient [35], for measuring severity of diabetes complications; Higher score means a more serious complication
Medical expenses	Annual outpatient expenses (LnY_8_)	Total amount of outpatient expenses incurred by a diabetes patient per year
	Annual inpatient expenses (LnY_9_)	Total amount of inpatient expenses incurred by a diabetes patient per year
	Annual medical expenses (LnY_10_)	Total amount of medical expenses incurred by a diabetes patient per year, which is equal to annual outpatient expenses plus annual inpatient expenses
	Annual out-of-pocket expenses (LnY_11_)	Expenses that are covered by the basic medical insurance but fall within the deductible and above the reimbursement cap; and expenses that fall above the deductible and below reimbursement cap but need to be borne by a diabetes patient
Expenditure of the basic medical insurance funds	Annual expenditure of the basic medical insurance fund on outpatient services (LnY_12_)	Total expenses paid by the basic medical insurance funds in the annual outpatient expenses incurred by a diabetes patient per year
	Annual expenditure of the basic medical insurance fund on inpatient services (LnY_13_)	Total expenses paid by the basic medical insurance funds in the annual inpatient expenses incurred by a diabetes patient per year
	Annual expenditure of the basic medical insurance fund on healthcare services (LnY_14_)	Total expenses paid by the basic medical insurance funds in the annual medical expenses incurred by a diabetes patient per year

##### Core explanatory variable and control variables

The core explanatory variable (X) of this study was the out-of-pocket expenses for outpatient care, which was expressed by the annual outpatient reimbursement ratio of a diabetes patient. A higher annual outpatient reimbursement ratio means a lower out-of-pocket expenses for outpatient care. The annual outpatient reimbursement ratio was equal to the annual expenditure of the basic medical insurance funds on outpatient services for a diabetes patient divided by his or her annual outpatient expenses.

Considering that age and other diseases suffered by diabetes patients may have an impact on health-seeking behaviors, health outcomes, medical expenses and expenditure of the basic medical insurance funds, the above relevant factors were included as control variables (U_i_) in this study. The core explanatory variable and control variables are shown in Table 2.

**Table 2:** Description of core explanatory variable and control variables

Variable Type	Variable	Definition
Core explanatory variable (X)	Annual outpatient reimbursement ratio	It is a percentage which is equal to the annual expenditure of the basic medical insurance funds on outpatient services for a diabetes patient divided by his or her annual outpatient expenses
Control variable (U_i_)	Age	——
	Nervous system disease	Whether a diabetes patient utilized any medical services because of nervous system disease within one year (Yes = 1, No = 0)
	Musculoskeletal system and connective tissue disease	Whether a diabetes patient utilized any medical services because of musculoskeletal system or connective tissue disease within one year (Yes = 1, No = 0)
	Circulatory system disease	Whether a diabetes patient utilized any medical services because of circulatory system disease within one year (Yes = 1, No = 0)
	Benign tumor	Whether a diabetes patient utilized any medical services because of benign tumor within one year (Yes = 1, No = 0)
	Malignant tumor	Whether a diabetes patient utilized any medical services because of malignant tumor within one year (Yes = 1, No = 0)
	Digestive system disease	Whether a diabetes patient utilized any medical services because of digestive system disease within one year (Yes = 1, No = 0)
	Eye and adnexal disease	Whether a diabetes patient utilized any medical services because of eye or adnexal disease within one year (Yes = 1, No = 0)
	Endocrine, nutritional and metabolic disease	Whether a diabetes patient utilized any medical services because of endocrine, nutritional or metabolic disease within one year (Yes = 1, No = 0)

## Results

### Health-seeking behaviors

The effect of the annual outpatient reimbursement ratio on a diabetes patient’s health-seeking behaviors is shown in Table 3. The correlation coefficients show that firstly, the annual outpatient reimbursement ratio has a significant positive effect on the annual number of outpatient visits and the annual number of medical visits. With each increase of 1% in the annual outpatient reimbursement ratio, the annual number of outpatient visits and the annual number of medical visits increased by 0.021 and 0.014, respectively. Secondly, there is a significant negative relationship between the annual outpatient reimbursement ratio and the annual number of hospitalizations. Each 1% increase in the reimbursement ratio is associated with a decrease of 0.006 in the annual number of hospitalizations.

**Table 3:** Effect of the annual outpatient reimbursement ratio on the number of a diabetes patient’s health-seeking behaviors

Variable	Annual number of outpatient visits	Annual number of hospitalizations		Annual number of medical visits
Annual outpatient reimbursement ratio	0.021**		−0.006***	0.014*
	(2.41)	(− 6.98)		(1.66)
Control variable	Y	Y		Y
Constant	2.886	−1.592***		1.294
	(0.62)	(− 3.16)		(0.27)
Individual fixed effects	Y	Y		Y
Time fixed effects	Y	Y		Y
R-squared	0.086	0.081		0.094
N	5,996	5,996		5,996
Observations	17,988	17,988		17,988

Note: The cluster robust standard errors are used in the estimation; *** p<0.01, ** p<0.05, * p<0.1

### Health outcomes

The empirical results in Table 4 show the effect of the annual outpatient reimbursement ratio on a diabetes patient’s health outcomes. It can be seen that increased annual outpatient reimbursement ratio significantly reduced the annual hospital stays and average length of a hospital stay. The annual length of hospital stays and average length of a hospital stay decreased by 1.2% and 1.1% respectively for every 1% increase in the annual outpatient reimbursement ratio. Additionally, the annual outpatient reimbursement ratio also has a significant negative impact on the number of diabetes complications and DCSI score. For each increase of 1% in the annual outpatient reimbursement ratio, the number of diabetes complications and DCSI score both decreased by 0.001.

**Table 4:** Effect of the outpatient reimbursement ratio on a diabetes patient’s health outcomes

Variable	Annual length of hospital stays	Annual average length of a hospital stay	Number of diabetes complications	DCSI score
Annual outpatient reimbursement ratio	−0.012***	−0.011***	−0.001***	−0.001***
	(− 12.11)	(− 12.57)	(− 5.55)	(− 4.65)
Control variable	Y	Y	Y	Y
Constant	−2.910***	−2.673***	1.923***	2.030***
	(− 4.56)	(− 4.71)	(13.61)	(11.29)
Individual fixed effects	Y	Y	Y	Y
Time fixed effects	Y	Y	Y	Y
R-squared	0.067	0.055	0.032	0.024
N	5,996	5,996	5,996	5,996
Observations	17,988	17,988	17,988	17,988

Note: The cluster robust standard errors are used in the estimation; *** p<0.01, ** p<0.05, * p<0.1

### Medical expenses

Table 5 presents that the annual outpatient reimbursement ratio has a significant negative effect on a diabetes patient’s medical expenses. According to the estimated results, the annual outpatient expenses, annual inpatient expenses, annual medical expenses and annual out-of-pocket expenses decreased by 2.2%, 4.6%, 2.6%, and 4.0% with every 1% increase in the annual outpatient reimbursement ratio, respectively.

**Table 5:** Effect of the annual medical reimbursement ratio on a diabetes patient’s medical expenses

Variable	Annual outpatient expenses	Annual inpatient expenses	Annual medical expenses	Annual out-of-pocket expenses
Annual outpatient reimbursement ratio	−0.022***	−0.046***	−0.026***	−0.040***
	(− 18.28)	(− 12.53)	(− 18.16)	(− 26.32)
Control variable	Y	Y	Y	Y
Constant	−2.136***	−11.661***	−2.941***	−1.490**
	(− 4.06)	(− 5.09)	(− 4.09)	(− 2.29)
Individual fixed effects	Y	Y	Y	Y
Time fixed effects	Y	Y	Y	Y
R-squared	0.178	0.061	0.159	0.226
N	5,996	5,996	5,996	5,996
Observations	17,988	17,988	17,988	17,988

Note: The cluster robust standard errors are used in the estimation; *** p<0.01, ** p<0.05, * p<0.1

### Expenditure of the basic medical insurance funds

The effect of the annual outpatient reimbursement ratio on the expenditure of the basic medical insurance funds for a diabetes patient is shown in Table 6. The results present that increase of annual outpatient reimbursement ratio significantly influences annual expenditure of the basic medical insurance funds on outpatient services and inpatient services. With each increase of 1% in the annual outpatient reimbursement ratio, the annual expenditure of basic medical insurance funds on outpatient services increased by 1.1%, and on inpatient services decreased by 4.4%. However, the annual outpatient reimbursement ratio has no significant effect on annual expenditure of the basic medical insurance funds on healthcare services.

**Table 6:** Effect of the annual outpatient reimbursement ratio on the expenditure of the basic medical insurance funds for a diabetes patient

Variable	Annual expenditure of the basic medical insurance fund on outpatient services	Annual expenditure of the basic medical insurance fund on inpatient services	Annual expenditure of the basic medical insurance fund on healthcare services
Annual outpatient reimbursement ratio	0.011***	−0.044***	−0.002
	(8.41)	(− 12.50)	(− 1.14)
Control variable	Y	Y	Y
Constant	−5.538***	−11.139***	−6.009***
	(− 10.05)	(− 5.09)	(− 6.83)
Individual fixed effects	Y	Y	Y
Time fixed effects	Y	Y	Y
R-squared	0.211	0.061	0.127
N	5,996	5,996	5,996
Observations	17,988	17,988	17,988

Note: The cluster robust standard errors are used in the estimation; *** p<0.01, ** p<0.05, * p<0.1

## Discussion

The results of this study indicated that decreasing the out-of-pocket expenses for diabetes outpatient care has a significant impact on enhancing the rationality of patients’ health-seeking behaviors. Previous researches have shown that the high out-of-pocket expenses for outpatient care not only inhibits patients from timely and appropriately seeking medical advice that could be considered as reasonable medical demands [36, 37], but also induces them to prefer inpatient services for more compensation from medical insurance, which is in the best interests of medical institutions [38]. According to the empirical results in this study, raising the outpatient reimbursement ratio for people with diabetes could markedly increase the number of outpatient visits and the total number of medical visits annually. That is to say, decreasing the out-of-pocket expenses for diabetes outpatient care could encourage patients to seek medical treatment actively. At the same time, thanks to the satisfaction of normal healthcare demands for outpatient services, their unreasonable requests for admission, caused by deficiency in reimbursement for outpatient services, might be reduced at least to some extent. It improves the rationality of health-seeking behaviors of people with diabetes finally.

In theory, for progressive diseases [39, 40], especially those chronic diseases requiring long-term healthcare services such as diabetes, raising the frequency of outpatient services usually leads to an increase in health outcomes [41]. Similar result is drawn from this study: after decreasing the out-of-pocket expenses for outpatient care, diabetes patients are more willing to make use of outpatient services, which could help to reduce the incidence or severity of diabetic complications and then significantly shorten annual length of hospital stays and average length of a hospital stay. Two implications behind this finding can be interpreted. For one thing, appropriate reimbursement for outpatient expenses will be instrumental in assisting diabetes patients to develop rational health-seeking behaviors, meaning these people will see a doctor if they feel sick or uncomfortable without hesitation or neglect [42]. This plays a key role in preventing the illness from becoming worse because it avoids delays in the treatment and people needing inpatient services for condition advancement continually. For another, since the number of outpatient visits increased, patients have more opportunities to acquire information and knowledge about their own diseases while doctors are able to better carry out disease management work and health guidance such as education on diet and sport, which makes great contribution to reducing the incidence or severity of diabetes complications and guarding against disease aggravation [43]. On account of these, people with diabetes gradually decrease the utilization of inpatient services and their health level ascend steadily.

As to the medical expenses, this study found raising the diabetes patients’ outpatient reimbursement ratio could notably reduce inpatient expenses and total expenses for individuals. It implied that getting benefit from patients’ rational health-seeking behaviors and better health status because of more access to outpatient care as above mentioned, part of original inpatient expenses will be substituted by the outpatient expenses, ultimately reducing total medical expenses. The out-of-pocket expenses of diabetes patients is also reduced in the meantime. These discoveries are similar to the results of previous studies [44, 45]. Besides this, the outpatient expenses decrease as well, which further verifies the positive role of outpatient services in disease prevention, health outcomes promotion, treatment effectiveness improvement and medical expenses reduction.

What’s more, decreasing the out-of-pocket expenses for outpatient care has a great influence on strengthening the efficiency of the basic medical insurance funds in China. Making use of diabetes patients’ real records in the medical insurance claim dataset, this study proved that adding actual compensation for diabetes outpatient care increases the expenditure of the basic medical insurance funds on outpatient services. Nevertheless, affected by transfer effect between outpatient and inpatient expenses and promotion in health outcomes, the funds expenditure on inpatient services will be saved instead, which fills the increased part in outpatient care properly [46]. In general, decreasing the out-of-pocket expenses for outpatient care among people with diabetes may not cause excessive expenditure of the basic medical insurance funds, in other words, the basic medical insurance system could always be running smoothly. But in such a case, the individual financial burden of diabetes patients gets relieved and their rationality of health-seeking behaviors and health status are also improved simultaneously, which help China’s basic medical insurance system give full play to safeguard health of the insured and promote the efficient allocation of medical resources effectively.

To ensure the results and analysis above-mentioned are stable and reliable, this paper continues to study the effect of decreasing the out-of-pocket expenses for outpatient care on health-seeking behaviors, health outcomes and medical expenses of different groups (See Additional file 1). On the whole, the results of stratified analysis are basically consistent with the results above-mentioned. By further analysis, it could be found that compared with males, females are more sensitive to change of the out-of-pocket expenses, which makes them take more advantage of outpatient services and reduce the demand for hospitalizations. Owing to this, females have better health outcomes and could save more expenditure. Besides this, decreasing the out-of-pocket expenses for outpatient care of old people who are over 65 with diabetes seems to have a greater effect. For example, with increase of 1% in the annual outpatient reimbursement ratio, their added annual number of outpatient visits is three times than those under 65 while annual number of hospitalizations reduced more significantly.

## Conclusion

In conclusion, decreasing the out-of-pocket expenses appropriately for outpatient care among people with diabetes could make patients have a more rational health-seeking behaviors, a better health status and a more reasonable medical expenses while the expenditure of the basic medical insurance funds is stable totally.

The positive findings on decreasing the out-of-pocket expenses for diabetes outpatient care in this paper are of great significance to China and other countries particularly those with relatively scarce medical resources. Firstly, this article systematically clarified the quantitative relationships between the outpatient reimbursement ratio and the health-seeking behaviors, health outcomes, and medical expenses of people with diabetes, as well as the expenditure of the basic medical insurance funds. It will be conductive for countries who implement a social health insurance system to make a more scientific and accurate decision when they design their own policies of decreasing the out-of-pocket expenses for diabetes outpatient services. Secondly, the good results of decreasing the out-of-pocket expenses for diabetes outpatient care provide evidence for other policies optimization. For example, these findings inspire us that it is necessary to eliminate the difference of out-of-pocket expenses for outpatient care between the local and nonlocal diabetes patients, which has a pivotal impact on cross-region healthcare services [47].

This study also has some limitations. First, only limited information is collected and included in the medical insurance claim dataset, resulting in some information such as type of diabetes and salary income are not included in this study. Second, the sample region is an area of developed economy in eastern China, so it needs a further study to prove whether these results apply to other areas. Third, although decreasing the out-of-pocket expenses for diabetes outpatient care will bring a lot of benefits, but how to avoid inducing the insured to overuse outpatient services still requires additional research to investigate.

## Electronic supplementary material

Below is the link to the electronic supplementary material.

Additional file 1 Effects of decreasing the out-of-pocket expenses for outpatient care on health-seeking behaviors, health outcomes and medical expenses of different groups.

## Data Availability

The datasets used and/or analysed during the current study are available from the corresponding author on reasonable request.

## Footnotes

1 The conversion rate on March 1, 2022 of 1 USD = 6.31 RMB is used in this article.
