# Supplementary material for: Effects of decreasing the out-of-pocket expenses for outpatient care on health-seeking behaviors, health outcomes and medical expenses of people with diabetes: evidence from China
Source: Int J Equity Health. 2022 Nov 16;21:162. doi: 10.1186/s12939-022-01775-5 (PMC9667616; doi:10.1186/s12939-022-01775-5)
Supplement: Supplementary file 1 — Additional file 1 Effects of decreasing the out-of-pocket expenses for outpatient care on health-seeking behaviors, health outcomes and medical expenses of different groups. [file 12939_2022_1775_MOESM1_ESM.docx]

**Additional file 1**

Table S1 Effects of decreasing the out-of-pocket expenses for outpatient care on health-seeking behaviors, health outcomes and medical expenses of different groups

| Variable | | Sex | | Age | |
| --- | --- | --- | --- | --- | --- |
|  |  | (1) Male | (2) Female | (3) Under 65 | (4) Over 65 |
| Health-seeking behaviors | Annual number of outpatient visits | 0.022  (1.55) | 0.021**  (2.01) | 0.010  (0.76) | 0.030***  (2.65) |
|  | Annual number of hospitalizations | -0.004***  (-3.11) | -0.008***  (-6.41) | -0.004***  (-3.29) | -0.008***  (-6.05) |
|  | Annual number of medical visits | 0.017  (1.23) | 0.014  (1.28) | 0.006  (0.46) | 0.022*  (1.95) |
| Health outcomes | Annual length of hospital stays | -0.011***  (-7.46) | -0.013***  (-9.49) | -0.008***  (-5.33) | -0.016***  (-10.98) |
|  | Annual average length of a hospital stay | -0.011***  (-8.06) | -0.012***  (-9.62) | -0.007***  (-5.52) | -0.014***  (-11.46) |
|  | Number of diabetes complications | -0.001***  (-3.76) | -0.001***  (-3.98) | -0.001***  (-4.36) | -0.001***  (-3.60) |
|  | DCSI score | -0.001***  (-2.90) | -0.001***  (-3.55) | -0.001***  (-3.72) | -0.001***  (-2.99) |
| Medical expenses | Annual outpatient expenses | -0.021***  (-10.73) | -0.022***  (-15.00) | -0.022***  (-11.12) | -0.021***  (-14.64) |
|  | Annual inpatient expenses | -0.043***  (-8.03) | -0.047***  (-9.60) | -0.029***  (-5.69) | -0.057***  (-11.24) |
|  | Annual medical expenses | -0.025***  (-10.97) | -0.027***  (-14.47) | -0.024***  (-10.21) | -0.028***  (-15.09) |
|  | Annual out-of-pocket expenses | -0.040***  (-16.93) | -0.040***  (-20.14) | -0.041***  (-15.93) | -0.039***  (-21.48) |
| Expenditure of the basic medical insurance funds | Annual expenditure of the basic medical insurance fund on outpatient services | 0.011***  (5.44) | 0.012***  (6.54) | 0.011***  (4.63) | 0.012***  (7.60) |
|  | Annual expenditure of the basic medical insurance fund on inpatient services | -0.041***  (-8.01) | -0.045***  (-9.56) | -0.027***  (-5.65) | -0.054***  (-11.22) |
|  | Annual expenditure of the basic medical insurance fund on healthcare services | -0.001  (-0.35) | -0.003  (-1.10) | 0.004  (1.26) | -0.005**  (-2.43) |
| N | | 2371 | 3625 | 2179 | 3817 |

Note: The cluster robust standard errors are used in the estimation; *** p＜0.01, ** p＜0.05, * p＜0.1.
